# Can stress predict delivery date?: Role of chronic and acute stress to the threatened preterm labor as predictors of delivery date

**DOI:** 10.1192/j.eurpsy.2021.1619

**Published:** 2021-08-13

**Authors:** J. Buesa Lorenzo, A. García-Blanco, M. Vento, A. Moreno-Giménez, L. Campos Berga, V. Diago, D. Hervás, C. Cháfer-Pericás, P. Sáenz González

**Affiliations:** 1 Psychiatry, University and Polytechnic Hospital La Fe, Valencia, Spain; 2 Neonatal Research Group, The Medical Research Institute Hospital La Fe (IIS La Fe), Valencia, Spain; 3 Gynaecology And Obstetrics, University and Polytechnic Hospital La Fe, Valencia, Spain; 4 Data Science Unit, Biostatistics, And Bioinformatics, The Medical Research Institute Hospital La Fe (IIS La Fe), Valencia, Spain

**Keywords:** stress, Threatened preterm labor, predictor

## Abstract

**Introduction:**

Threatened preterm labor (TPL) is a traumatic event during pregnancy that involves a threat to the physical integrity of the upcoming baby. Despite biomarkers would be the strongest delivery date predictors, an assessment of chronic and acute stress response to TPL diagnosis may improve this prediction.

**Objectives:**

The objective is to predict delivery date in women with TPL based on their response to this diagnosis and chronic stressors, along with relevant obstetric variables.

**Methods:**

A prospective cohort study was conducted with a sample was formed by 157 pregnant women with TPL diagnosis between 24 and 31 weeks. Determination of salivary cortisol, α-amylase levels, along with anxiety and depression symptoms were measured to estimate stress response to TPL. Cumulative life stressors as traumas, social and familiar functioning were also registered. To examine the effect of the possible predictor variables of delivery date, linear regression models were used.

**Results:**

A correlation was found between the variables of response to chronic stress and between the variables of psychological response to stress. The main predictors of preterm delivery were low family adaptation, higher BMI, higher cortisol levels, and the week of diagnosis of TPL (<29 weeks of gestation).

**Conclusions:**

The best predictor of delivery date was the combination of the stress response to the diagnosis of TPL measured by cortisol in saliva, cumulative life stressors (mainly family adaptation) and obstetric factors (week TPL and BMI). Through psychosocial therapeutic intervention programs, it is possible to influence this modifiable predictive factors of preterm birth in symptomatic women.

